# 482. The modern face of esophageal candidiasis (EC) in an oncology center: Analysis of 323 cancer patients with EC

**DOI:** 10.1093/ofid/ofac492.540

**Published:** 2022-12-15

**Authors:** Takahiro Matsuo, Ben S Singh, Sebastian Wurster, Ying Jiang, Manoop S Bhutani, Dimiitrios P Kontoyiannis

**Affiliations:** The University of Texas MD Anderson Cancer Center, Houston, Texas; The University of Texas MD Anderson Cancer Center, Houston, Texas; The University of Texas MD Anderson Cancer Center, Houston, Texas; The University of Texas MD Anderson Cancer Center, Houston, Texas; The University of Texas MD Anderson Cancer Center, Houston, Texas; The University of Texas MD Anderson Cancer Center, Houston, Texas

## Abstract

**Background:**

Historically, EC had been a common infection in the pre-azole era, and it was associated with thrush, cytopenia, chemotherapy, and debility in patients (pts) with malignancy. However, the presentation and outcomes of EC in cancer pts in the current era of new cancer treatments and frequent use of azoles as antifungal prophylaxis are scarcely studied.

**Methods:**

We retrospectively reviewed the risk factors, clinical and laboratory features, and outcome of pathology-documented EC in pts at MD Anderson Cancer Center from January 2017 to October 2021. We followed established criteria for the endoscopy grade of EC (B E Kodsi, et al. Gastroenterology. 1976;71(5):715-9). We used a binary multivariable logistic regression model to determine independent risk factors for treatment failure (defined as relapse within 1 year from EC diagnosis) among pts who received fluconazole for EC treatment.

**Results:**

Among 323 pts with EC, 290 pts (90%) had solid tumors; only 33 pts (10 %) had hematological malignancies. Only 7 (2%) of pts had prior antifungal prophylaxis Esophageal cancer was most common (35%), followed by lung cancer (10.8%), gastric cancer (10.2%), and colon cancer (5.9%). 229 pts (71%) received chemotherapy within 30 days prior to EC diagnosis. The most common symptoms were dysphagia (39%), nausea (20%), and odynophagia (15%); 33% of EC pts were asymptomatic. Oral thrush (2%) was uncommon. Most patients (83%) had non-ulcerative EC (endoscopic grade 1-2), 13% had grade 3, and 4% had grade 4 EC. 206 pts (64%) received antifungal treatment (oral fluconazole in 202). Among pts treated with fluconazole, 27 (13%) had treatment failure. Underlying esophageal disease was the only independent predictor of fluconazole treatment failure on multivariate analysis (adjusted odds ratio 3.88, 95%CI 1.49-10.1).

**Conclusion:**

In a large cohort of 323 contemporary pts with EC the entity is predominantly encountered in pts with solid tumor on no antifungal prophylaxis, especially the ones with underlying esophageal pathology which is a predictor of azole treatment failure. Concomitant thrush was uncommon, and in 1/3 of pts EC was only an endoscopic finding.

**Disclosures:**

**Dimiitrios P. Kontoyiannis, MD, ScD, PhD (hon)**, AbbVie: Advisor/Consultant|Astellas Pharma: Advisor/Consultant|Astellas Pharma: Grant/Research Support|Astellas Pharma: Honoraria|Cidara Therapeutics: Advisor/Consultant|Gilead Sciences: Advisor/Consultant|Gilead Sciences: Grant/Research Support|Gilead Sciences: Honoraria|Merck: Advisor/Consultant.

